# *Glossogyne tenuifolia* (*Hsiang-ju*) extract suppresses T cell activation by inhibiting activation of c-Jun N-terminal kinase

**DOI:** 10.1186/s13020-017-0130-4

**Published:** 2017-04-11

**Authors:** Jer-Yiing Houng, Tzong-Shyuan Tai, Shu-Ching Hsu, Hsia-Fen Hsu, Tzann-Shun Hwang, Chih-Jiun Lin, Li-Wen Fang

**Affiliations:** 1 Department of Nutrition, I-Shou University, No.8, Yida Rd., Yanchao District, Kaohsiung City, 82445 Taiwan; 2 Department of Medical Research, E-Da Hospital, Kaohsiung City, 82445 Taiwan; 3 School of Medicine for International Students, I-Shou University, Kaohsiung City, 82445 Taiwan; 4 National Institute of Infectious Diseases and Vaccinology, NHRI, Miaoli County, 35053 Taiwan; 5 Department of Medical Research, Show-Chwan Memorial Hospital, Changhua County, 50008 Taiwan; 6 Graduate Institute of Biotechnology, Chinese Culture University, Taipei City, 11114 Taiwan; 7 Department of Leisure and Recreation Management, Da-Yeh University, Changhua County, 51591 Taiwan

## Abstract

**Background:**

*Glossogyne tenuifolia* (GT) (*Hsiang-ju*) is a Chinese herbal medicine previously exhibited an anti-inflammatory activity. This study aimed to investigate the effect of GT ethanol extract (GTE) on T cell-mediated adaptive immunity.

**Methods:**

Human peripheral blood mononuclear cells (PBMCs) and Jurkat T cells were activated by phytohemagglutinin in the presence of various doses (3.13–50 μg/mL) of GTE. The effect of GTE on T cell activation was examined by a proliferation assay of activated PBMCs and the level of the activation marker CD69 on the surface of activated Jurkat T cells. Apoptosis was determined by propidium iodide staining in hypotonic solution. Signaling pathway molecules were assessed by western blotting.

**Results:**

*Glossogyne tenuifolia* ethanol extract was demonstrated to inhibit T cell activation, not only in the proliferation of human PBMCs at the concentrations of 12.5, 25 and 50 μg/mL (*P* = 0.0118, 0.0030 and 0.0021) but also in the CD69 expression in Jurkat cells, which was not due to the cytotoxicity of GTE. The presence of GTE did not change the activity of nuclear factor kappa-light-chain-enhancer of activated B cells or extracellular signal-regulated kinase upon T cell activation. In addition, GTE significantly reduced activation of c-Jun N-terminal kinase (JNK) (*P* = 0.0167) and p38 (*P* = 0.0278). Furthermore, decreased JNK activation mediated the preventive effect of GTE on T cell activation-induced cell death (AICD).

**Conclusion:**

*Glossogyne tenuifolia* ethanol extract inhibited T cell activation of Jurkat cells and freshly prepared human PBMCs due to suppression of JNK activity. Furthermore, GTE inhibited AICD by blocking prolonged JNK phosphorylation in activated T cells. Taken together, the anti-inflammatory effects exerted by GTE were mediated via suppression of JNK phosphorylation in T cell activation.

**Electronic supplementary material:**

The online version of this article (doi:10.1186/s13020-017-0130-4) contains supplementary material, which is available to authorized users.

## Background

Inflammation is a normal host defense mechanism against infection, involving not only innate but also adaptive immunity. However, uncontrolled inflammation may lead to tissue damage and cause autoimmune or autoinflammatory diseases. In autoinflammatory diseases, such as type 2 diabetes and obesity, macrophages in innate immunity become dysfunctional [1]. In contrast, autoimmune diseases, such as rheumatoid arthritis, inflammatory bowel disease, type 1 diabetes, psoriasis, lupus, and multiple sclerosis, are often mediated by lymphocytes of adaptive immunity rather than macrophages [1]. T cells are the dominant dysfunctional cells or initiators during the development of autoimmune diseases [1].

Anti-inflammatory drugs have several adverse side effects. *Glossogyne tenuifolia* (GT) (*Hsiang-ju*) is a perennial herb of *Asteraceae* that is distributed from south Asia to Australia. GT is a Chinese medicine used in antipyretic, hepatoprotective, and anti-inflammatory remedies [2, 3]. Previous studies have shown that GT extract possesses pharmacological activities of antioxidation [4–6], cytotoxicity against several cancer cell lines [4, 7], protection against endothelial cell injury [8], and prevention of osteoclast-related diseases such as osteoporosis [9]. In addition, GT exhibited activity in immunomodulation [10–12]. GT extract downregulated the gene expression of inducible nitric oxide synthase and cyclooxygenase-2, as well as the production of proinflammatory cytokines upon stimulation by lipopolysaccharide (LPS) in Raw 264.7 cells [10] and human peripheral blood mononuclear cells (PBMCs) [11]. However, the effect of GT on adaptive immune cells, such as T cells, remains unclear.

Currently, the discovery of safe natural products for the treatment of inflammatory disorders focuses on inhibition of macrophage activity [13]. However, cells of the adaptive immune system, such as T and B cells, mediate inflammatory processes of some inflammatory diseases such as rheumatoid arthritis and inflammatory bowel disease. Molecules of the activation pathway of T cells might be a good target for modulation of these inflammatory diseases [1]. Resting T cells are in the G0 phase of the cell cycle. In response to T cell receptor (TCR) ligation, mitogenic stimulation, or a combination of a phorbol ester and calcium ionophore, T cells activate and transduce activation signals, leading to their proliferation. TCRs initiate signaling cascades that lead to activation of downstream mitogen-activated protein kinases (MAPKs) and nuclear factor kappa-light-chain-enhancer of activated B cells (NF-κB) [14]. Extracellular signal-regulated kinase (ERK), c-Jun N-terminal kinase (JNK) and p38 MAPK are important MAPKs involved in T cell activation. Cell proliferation and differentiation into effector cells as well as the production of interleukin (IL)-2 reflect the degree of T cell activation, and a lack of either MAPK or NF-κB impairs T cell activation-induced proliferation and IL-2 production [15].

Activated T cells undergo apoptosis upon re-stimulation, which is called activation-induced cell death (AICD). AICD is a critical mechanism to eliminate autoreactive lymphocytes in the immune system and maintain immune tolerance and homeostasis [16]. During cancer progression, the immune system acts as a significant barrier. Immune cells, such as CD8^+^ cytotoxic T lymphocytes (CTLs), CD4^+^ helper T cells, and natural killer cells, contribute to immunosurveillance and tumor elimination [17]. In antitumor immunotherapy, AICD of T cells is believed to be the key event leading to the failure of anti-tumor effects [18, 19]. Several studies of cancer immunotherapy using different approaches have highlighted inhibition of AICD to rescue activated T cells, thereby enhancing anti-tumor immune responses [18–21]. In adoptive cancer immunotherapy [22], tumor antigen-specific T cells are susceptible to AICD upon encountering the tumor antigen, causing the low success rate of cancer immunotherapy. Phosphorylation of JNK is critical for AICD of melanoma antigen-specific primary CTLs, and blocking JNK activation prevents AICD of CTLs [20, 23, 24]. This study aimed to investigate the effect of GT ethanol extract (GTE) on T cell-mediated adaptive immunity.

## Methods

### Preparation of GTE

GT plant materials were purchased from an herb store in Penghu Island, Taiwan, and deposited as the number of ISU-JYH-001 in the Herbarium of I-Shou University (Kaohsiung City, Taiwan). The nucleotide sequences of internal transcribed spacer (ITS) 1, ITS2, and 5.8S rDNA were isolated from this plant, and the species was confirmed by the NCBI DNA database [4]. Dry whole plant materials were ground into powder. GT (5.3 kg) was extracted with 20 L of 95% ethanol at room temperature for 1 day and repeated for three times. The extracted solutions were filtered through medicinal gauze. The GTE was obtained by removing the solvent with a rotary evaporator (VP-60DJ, Panchum Co., Kaohsuing City, Taiwan) and drying in a freeze drier (FD8530, Panchum Co.). The total dry weight of this extract was 777 g, and the extraction yield was 14.7%. The quality of the herb extract was monitored by high performance liquid chromatography (L-7100, Hitachi, Tokyo, Japan) analysis using luteolin and luteolin-7-glucoside (Sigma Chemicals Co., St Louis, MO, USA) as the principle control standards [4, 9]. The dry GTE was dissolved in dimethyl sulfoxide (DMSO) at the concentration 50 mg/mL as the GTE stock. The final GTE concentrations (3.13, 6.3, 12.5, 25 and 50 μg/mL) were made by culture medium dilution. The DMSO was added no more than 0.1% (v/v) and used as a mock control in this study.

### Cell culture and human PBMC preparation

Human PBMCs were prepared from the whole blood and human leukemia cell line Jurkat was obtained from American Type Cell Collection (ATCC, Manassas, VA, USA). Human PBMCs and leukemic Jurkat cells were grown in RPMI-1640 medium (GE Healthcare, Piscataway, NJ, USA) with 10% fetal bovine serum (FBS; Thermo Fisher Scientific Inc., Waltham, MA, USA), 1% penicillin/streptomycin (Sigma-Aldrich, St. Louis, MO, USA), and 2 mM l-glutamine (Sigma-Aldrich) at 37 °C in a humidified atmosphere with 5% CO_2_. Human PBMCs were isolated from whole blood using a Ficoll-Paque plus density gradient [25]. Whole bloods from healthy volunteers were mixed with the same volume of Hank’s balanced salt solution (HBSS; Thermo Fisher, St. Louis, MO, USA). The blood-HBSS mixture was layered over Ficoll-Paque PLUS (GE Healthcare) 1:1 (v/v) and centrifuged (5810R, Eppendorf, Hamburg, Germany) (400×*g*) for 20 min at 25 °C with the break off. The PBMC layer at the interface was collected and washed with HBSS three times by centrifugation (300×*g*) for 5 min at 4 °C. The PBMCs were suspended in RPMI-1640 medium. The study protocols were approved by the Institutional Review Board of the Research Ethics Committee of National Health Research Institutes (EC1001101) (Additional file 1).

### Western blotting

For each sample, 1 × 10^6^ cells were washed with phosphate buffered saline (PBS; pH 7.2) and lysed in freshly prepared cell lysis buffer (20 mM Tris–HCl, pH 7.5, 150 mM NaCl, 1 mM Na_2_EDTA, 1 mM EGTA, 1% Triton X-100, 2.5 mM sodium pyrophosphate, 1 mM β-glycerophosphate, 1 mM Na_3_VO_4_, 1 µg/mL leupeptin, and 1 mM phenylmethanesulfonyl fluoride). The cell lysate was separated from debris by centrifugation at 13,200×*g* for 10 min at 4 °C. The protein concentration was measured by Bio-Rad Protein Assay Dye Reagent (Bio-Rad Laboratories, Hercules, CA, USA). An aliquot of 20 μg protein was separated on a 10% polyacrylamide gel and transferred onto a polyvinylidene fluoride membrane (EMD Millipore, Billerica, MA, USA). The membranes were subsequently blocked with 5% dry skim milk in Tris-buffered saline (TBS; pH 7.5). The membranes were hybridized with the indicated primary antibody at 4 °C overnight, washed three times with 0.05% TBST solution (50 mM Tris–Cl, pH 7.5, 150 mM NaCl, and 0.05% Tween-20) for 10 min, and then hybridized with a horseradish peroxidase (HRP)-conjugated secondary antibody at room temperature for 1 h. After washing three times with 0.05% TBST for 10 min each, the membrane was incubated with Luminata™ Western HRP Substrates (EMD Millipore). The signals were detected using Image Lab software Version 2.0 (Bio-Rad Laboratories).

### T cell proliferation and activation assay

PBMCs (1 × 10^5^) were pretreated with GTE (3.13, 6.3, 12.5, 25 and 50 μg/mL) for 30 min and then activated by 2.5 μg/mL phytohemagglutinin (PHA) in each well of a 96-well plate for 48 h. An aliquot of 0.5 μCi ^3^H-thymidine was added to each well. After 20 h of incubation, the cells were harvested with a FilterMate 96-well harvester (PerkinElmer Inc, Waltham, MA, USA) for a proliferation assay. The radioactivity (counts per minute, CPM) was measured to quantify cell proliferation with a microplate scintillation and luminescence counter (TopCount NXT, Packard Instrument Co, Meriden, CT, USA). Jurkat cells (1 × 10^5^) were pretreated with GTE (25 μg/mL) for 30 min and then stimulated by 10/80 ng/mL 12-*O*-tetradodecanoyl-phorbol-13-acetate (PMA)/ionomycin (P/I) in each well of a 96-well plate for 24 h. Cells were then harvested for surface marker staining. To detect NF-κB activation, phosphorylation of nuclear factor of kappa light polypeptide gene enhancer in B-cells inhibitor (IκB) and p65 proteins were determined by western blotting. Jurkat cells (1 × 10^6^) were pretreated with GTE (25 μg/mL) for 30 min and then stimulated by PHA (2.5 μg/mL) for 0, 1, and 3 h. Cell lysates were then prepared for western blot analysis. To detect MAPK activation (p-ERK, p-p38, and p-JNK), 1 × 10^6^ Jurkat cells were pretreated with GTE (25 μg/mL) for 30 min and then stimulated with 2.5 μg/mL PHA for 0, 10 and 20 min. Cell lysates were then prepared for western blotting. Primary antibodies against p-IκB (#2859, 1:500), p-p65 (#3033, 1:2000), p-ERK (#9101, 1:3000), p-p38 (#4511, 1:1000), and p-JNK (#4671, 1:500) were purchased from Cell Signaling Technology (Beverly, MA, USA). Primary antibodies against α-tubulin (#2871, 1:3000) was purchased from Epitomics Inc. (Burlingame, CA, USA). Primary antibodies against ERK2 (sc-154, 1:5000), p38 (sc-535, 1:3000), and JNK1 (sc-1648, 1:1000), and an HRP-conjugated secondary antibody were purchased from Santa Cruz Biotechnology (Dallas, TX, USA).

### Cell surface staining

Jurkat cells (1 × 10^5^) were stimulated with P/I (10/80 ng/mL) for 24 h. The cells were then stained with CD69-PE (12-0699, 1:50, eBioscience, San Diego, CA, USA) in staining buffer (PBS with 1% FBS) on ice for 20 min and then washed three times with staining buffer. The CD69-positive population was evaluated by a flow cytometer (FACSCalibur; BD Biosciences). Data were analyzed with FlowJo software (BD Biosciences). The signal obtained for the cells treated without or with GTE alone and stained with mouse IgG1 isotype control-PE (12-4714, 1:50, eBioscience) was used as a control respectively.

### Apoptosis assay

Apoptosis was determined by propidium iodide (PI) (Sigma-Aldrich) staining [26]. PBMCs or Jurkat cells (1 × 10^5^) were treated with various concentrations of GTE (3.13, 6.3, 12.5, 25 and 50 μg/mL) in each well of a 96-well plate for 24 h. Harvested cells were suspended in a hypotonic solution (0.1% sodium citrate, 0.1% Triton X-100, and 20 μg/mL PI). The extent of the sub-G0/G1 peak representing apoptotic cells was measured by gating on cells stained in the region below the G0/G1 peak. The apoptotic sub-G0/G1 population was examined by flow cytometry and analyzed with FlowJo software (BD Biosciences).

### AICD assay

Healthy female Balb/c mice were obtained from National Laboratory Animal Center (NLAC, Taipei, Taiwan) at 4 weeks of age. The animals were maintained in cages (5 mice/cage) under standard conditions. The mice were euthanized by use of CO_2_ exposure. CD4^+^ T cells isolated from the splenocytes of C57BL/6 mice were activated by antibodies against CD3 (16-0031, eBioscience) and CD28 (16-0281, eBioscience) (1 and 2 μg/mL, respectively) in the presence of 100 U/mL IL-2 for 5 days. The activated T cells were washed three times and reactivated by the anti-CD3 (16-0031, eBioscience) antibody (0.5 μg/mL) for 8 h. The cells were then harvested for an apoptosis assay. All animal experiments were performed in accordance with protocols approved by the Institutional Animal Care and Use Committee of I-Shou University (IACUC-ISU-96002) (Additional file 2). The combination of P/I and sodium orthovanadate (Na_3_VO_4_) induces significant AICD in Jurkat T cells [27]. Jurkat cells (1 × 10^5^) were pretreated with GTE (25 μg/mL) for 30 min and then activated by P/I (10/80 ng/mL) and 1.5 mM Na_3_VO_4_ in each well of a 96-well plate for 24 h. The cells were then harvested for an apoptosis assay.

### Statistical analyses

Each experiment was performed at least three times and measured in triplicates. The data were presented as mean ± standard deviation (SD). Each GTE-treated group was compared to the respective mock-treated (DMSO) control group. Statistical differences were analyzed by Student’s *t*-test (*P < 0.05, **P < 0.01 and ***P < 0.001). Data were analyzed using GraphPad Prism software (GraphPad Software, La Jolla, CA, USA).

### Information of experimental design and resources

Details of our experimental design and statistics and all resources used in this study were included in Additional file 3.

## Results

### Inhibitory effects of GTE on the activation of T cells and PBMCs

Mitogen stimulation hydrolyzes membrane polyphosphoinositides to inositol trisphosphate (IP3) and diacylglycerol (DAG). Then, IP3 releases Ca^2+^ into the cytosol, and DAG activates protein kinase C (PKC). These synergistic effects lead to progression of the cell cycle and proliferation of T cells [28, 29]. A combination of phorbol esters, which activates PKC, and a calcium ionophore, which increases the intracellular Ca^2+^ concentration, mimic the signals triggered by the TCR or mitogen stimulation and can be used as agents for T cell activation. In addition, CD69 is an early activated surface molecule on T cells, and expression of CD69 has been frequently used as an indicator for T cell activation [30].

**Fig. 1:**
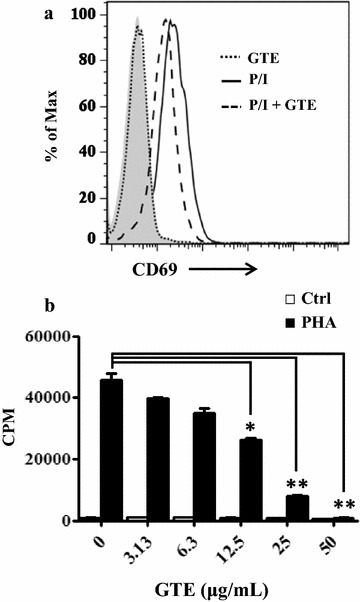
GTE blocks T cell activation. **a** CD69 induction decreased in GTE-treated Jurkat T cells. Jurkat T cells were activated by PMA/ionomycin alone (P/I;* solid line*) or with 25 μg/mL GTE (*long dashed line*). GTE treatment alone (*short dashed line*) was used as a control. The cells were stained with an anti-CD69-PE antibody. The CD69-positive cells were then analyzed by flow cytometry. Data were assessed with FlowJo software. **b** Decreased proliferation of GTE-treated PBMCs. Freshly purified PBMCs were pretreated with various doses of GTE for 30 min and then stimulated by 2.5 μg/mL PHA. Cell proliferation was examined by ^3^H-thymidine incorporation after a 20 h pulse with 0.5 μCi/well ^3^H-thymidine. Data are expressed as counts per minute (CPM) of ^3^H-thymidine uptake. A significant difference from the vehicle is indicated as *P < 0.05 or **P < 0.01

GTE has been reported to exert an inhibitory effect on LPS-treated macrophage and human PBMC activation [10, 11]. To further explore the influence of GTE on adaptive immunity, we examined the effect of GTE on the activation of T cells. PHA (a mitogen) or a combination of PMA (a phorbol ester) and ionomycin (a calcium ionophore) were used to activate T cells. First, Jurkat cells were pretreated with 25 μg/mL GTE for 30 min and then P/I were added to induce T cell activation for 24 h. The expression of CD69 was used as the T cell activation marker [31]. Figure 1a shows that GTE treatment reduced the fluorescence intensity of CD69-PE on P/I-activated Jurkat cells. The mean fluorescence intensity (MFI) was determined after analyzing 10,000 cells. The MFI changes of GTE and mock control were expressed as the fold changes relative to the individual group without P/I stimulation. The relative MFI fold changes of GTE-treated and mock control were 2.69 ± 0.01 and 4.14 ± 0.12 respectively. These data demonstrated that GTE administration decreased P/I-induced CD69 expression on P/I-activated Jurkat cells (*P* = 0.0044). In addition, the proliferation of primary T cells, representing successful activation of T cells, was used to evaluate T cell activation of PHA-stimulated PBMCs. Proliferation of PBMCs triggered with PHA (2.5 μg/mL) was suppressed by GTE/PHA cotreatment compared with PHA treatment alone at the GTE concentrations of 12.5, 25 and 50 μg/mL (*P* = 0.0118, 0.0030 and 0.0021) (Fig. 1b). The results of ^3^H-thymidine incorporation of PHA-activated PBMCs from four individual donors showed the suppressive trend of GTE in a dose-dependent manner (Additional file 4). The inhibitory effect of GTE on CD69 expression of activated Jurkat T cells (Fig. 1a) correlated well with the proliferation of PHA-stimulated human PBMCs in which GTE inhibited PHA-induced proliferation by up to 80% at 25 μg/mL (Fig. 1b). These results indicated that the GTE inhibited activation of both Jurkat T cells and human PBMCs.

### Cytotoxic effect of GTE on Jurkat T cells and PBMCs

**Fig. 2:**
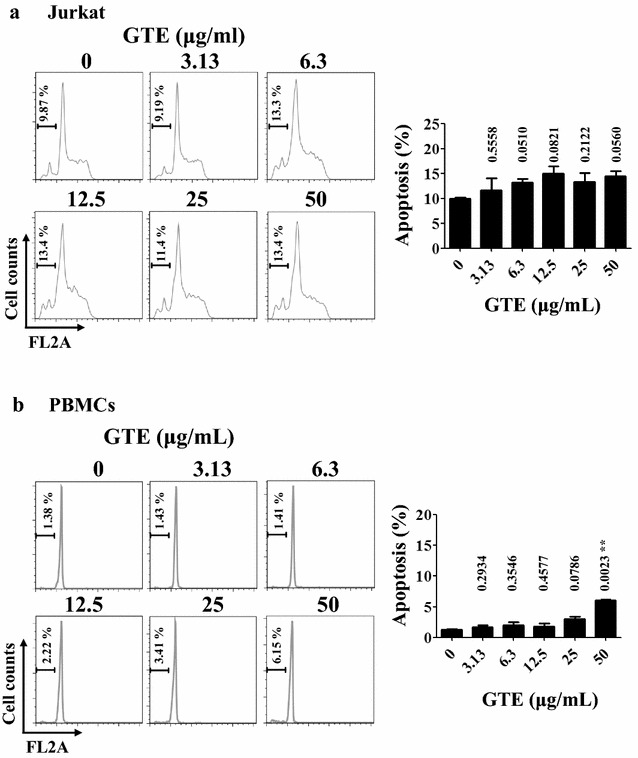
GTE does not induce apoptosis of Jurkat cells or PBMCs. Jurkat cells (**a)** and PBMCs (**b**) were treated with GTE at the concentrations indicated for 24 h. The cells were stained with 20 μg/mL propidium iodide (PI) in a hypotonic solution (0.1% sodium citrate and 0.1% Triton X-100). The apoptotic cells were then analyzed by flow cytometry. Data were analyzed with FlowJo software. The *left panel* is the original apoptosis data. The *right panel* is quantitative data. The extent of the sub-G0/G1 peak representing apoptotic cells was measured by gating on cells in the region below the G0/G1 peak. Numbers above the *bar* in the *right panel* of Jurkat cells (**a**) and PBMCs (**b**) are *P*-values relative to the control group. A significant difference from the control is indicated as **P < 0.01

Cytotoxic activity of GTE has been reported in certain cancer cell lines such as HepG2, Hep3B, MDA-MB-231, and MCF-7 [4, 7, 32]. This study showed that the death of cancer cells was mediated through the apoptotic pathway. The downregulating effect of GTE on T cell activation could be due to a cytotoxic effect on T cells. Thus, to determine whether GTE has a cytotoxic effect on T cells, Jurkat cells were cultured in the presence of various doses of GTE for 24 h, and then the degree of apoptosis was examined. Figure 2a shows minimal apoptosis in Jurkat cells (<20%) treated with GTE at up to 50 μg/mL. In addition, almost no apoptosis was observed in PBMCs treated with GTE at less than 25 μg/mL (Fig. 2b). Because GTE did not induce apoptosis of human PBMCs at 25 μg/mL, this dosage was used for the following activation assay. These results suggested that the inhibitory effect of GTE on T cell activation was not due to a cytotoxic effect.

### GTE down-regulated JNK phosphorylation, but not NF-κB activities, in activated human T cells

**Fig. 3:**
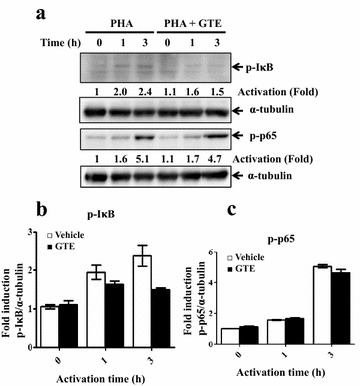
NF-κB activation is normal upon treatment with GTE. **a** Jurkat T cells were activated by PHA or PHA with GTE. Protein extracts were prepared at the indicated time points and subjected to western blot analysis. p-IκB and p-p65 protein levels were detected, and α-tubulin was used as an internal control. The densities of protein bands were determined by Image Lab software. Densitometric quantification of each protein band was normalized by the internal control. The levels of p-IκB and p-p65 are normalized by α-tubulin. Data represented fold activation from the non-activated (0 h) density (**b**, **c**)

The T cell activation signal activates multiple downstream molecules including phosphorylation of MAPKs and NF-κB. It has been shown that inhibition of the DNA-binding activity of NF-κB upon PHA stimulation is significant at a high dosage of GTE (up to 150 μg/mL) but insignificant at a low GTE dose (less than 100 μg/mL) [12]. In this study, we further examined GTE effects on NF-κB activity in T cell activation signaling. In the NF-κB activation pathway, IκB is phosphorylated and undergoes degradation upon PHA stimulation. We therefore evaluated IκB phosphorylation upon T cell activation. Figure 3 shows that GTE-treated Jurkat cells displayed similar levels of IκB phosphorylation upon PHA stimulation. The phosphorylation of RelA/p65, one of the NF-κB members, regulates NF-κB transcriptional activity [33]. We found that RelA/p65 phosphorylation was normal after co-treatment with GTE (Fig. 3). These data suggest that the deficiency of T cell activation in the presence of GTE is independent of the NF-κB pathway.

**Fig. 4:**
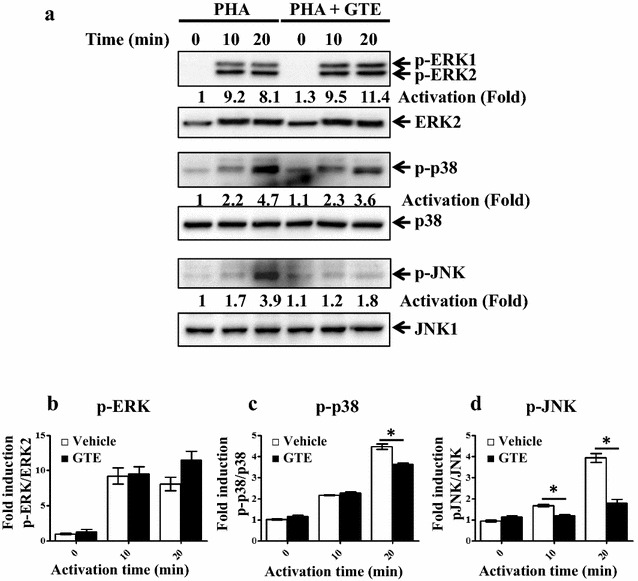
GTE attenuates JNK and p38 activation. **a** Jurkat T cells were activated by PHA alone or with GTE. Protein extracts were prepared at the indicated time points and subjected to western blot analysis. Activation of ERK, JNK and p38 was measured by phosphorylation of ERK, JNK, and p38. ERK2, JNK1, and p38 served as internal controls. Densitometric quantification of fold inductions of the levels of **b** p-ERK2 relative to ERK2, **c** p-p38 relative to p38, and **d** p-JNK relative to JNK1 protein. The densities of protein bands were determined by Image Lab software. Densitometric quantification of each protein was normalized by the internal control. The level of p-ERK2 was normalized by ERK2. The level of p-p38 was normalized by p38, and the level of p-JNK was normalized by JNK1. Data represented fold activation from the non-activated (0 h) density (**b**–**d**). A significant difference from the control is indicated as *P < 0.05

Because NF-κB signaling was normal in GTE-treated Jurkat cells, we mapped the signal defect in these cells. In addition to NF-κB signaling, the activities of MAPKs are important for T cell activation. Thus, we further examined the activity of three major MAPKs, ERK, p38 MAPK, and JNK, after stimulation. T cell activation increased ERK phosphorylation, and GTE administration did not alter the phosphorylation status of ERK (Fig. 4a, b). GTE slightly inhibited p38 MAPK phosphorylation after stimulation with PHA for 20 min (*P* = 0.0278) (Fig. 4a, c). Notably, JNK phosphorylation was significantly suppressed by GTE in activated Jurkat cells at the activation time 10 and 20 min (*P* = 0.0217 and 0.0167) (Fig. 4a, d). Taken together, the suppression of T cell activation by GTE might be attributed to a slight reduction in p38 activation and profound JNK activation.

### GTE attenuated prolonged JNK-induced AICD

**Fig. 5:**
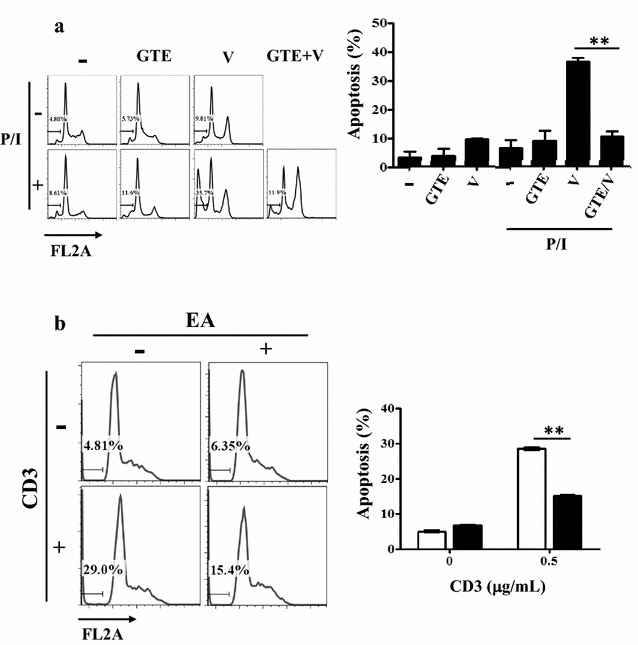
GTE blocks prolonged JNK-induced AICD. **a** Jurkat cells were treated with GTE, sodium orthovanadate (V; 1.5 mM), and/or PMA/ionomycin (P/I; 50/80 ng/mL) for 24 h. The cells were collected and stained with PI in a hypotonic solution. Apoptotic cells were analyzed by flow cytometry. Data were assessed with FlowJo software. **b** CD4 T cells were isolated from mouse spleen, activated with anti-CD3/CD28 antibodies, and cultured in the presence of IL-2. The activated T cells were re-activated by 0.5 μg/mL anti-CD3 antibody at day 5 and then harvested for an apoptosis assay. The *left panel* is the original apoptosis data. The *right panel* is quantitative data. The extent of the sub-G0/G1 peak representing apoptotic cells was measured by gating on cells in the region below the G0/G1 peak. A significant difference from the control is indicated as **P < 0.01

JNK is activated by a wide array of stress conditions and mitogenic stimulation. Previous studies have shown that JNK plays an important role in AICD [27]. It is important to examine whether GTE regulates AICD by modulating the activity of JNK. Na_3_VO_4_, a tyrosine phosphatase inhibitor, has been used to prolong JNK activation during T cell activation, whereas the combination of P/I and Na_3_VO_4_ upon T cell stimulation induces significant AICD in Jurkat T cells [27]. In this study, Na_3_VO_4_ was also shown to induce apoptosis upon T cell activation by P/I. Furthermore, administration of GTE with Na_3_VO_4_ or P/I did not induce apoptosis of Jurkat T cells (Fig. 5a). Figure 5a also demonstrates that GTE blocked Na_3_VO_4_-induced apoptosis in activated Jurkat T cells (*P* = 0.0037). Next, we examined the effect of GTE on AICD in reactivated T cells ex vivo. Naïve CD4 T cells were activated with anti-CD3/CD28 antibodies and maintained in IL2-containing medium for 5 days. The activated CD4 T cells were then re-activated with the anti-CD3 antibody to trigger AICD. Figure 5b shows that T cells underwent AICD through repeated stimulation of CD3, and GTE inhibited AICD of primary isolated T cells (*P* = 0.0016). These results suggested that GTE might attenuate prolonged JNK-induced AICD.

## Discussion

Several studies have investigated the anti-inflammatory mechanism of GTE, but all of them have focused on GTE effects in activated myeloid lineage macrophages [10, 11]. However, the inflammatory effect of GTE on T cells is still unknown. Cells of the adaptive immune system, especially T cells, mediate the tissue damage of some inflammatory diseases such as rheumatoid arthritis and inflammatory bowel disease [1]. In the present study, we found that GTE suppressed T cell activation in both Jurkat T cells and in human PBMCs (Fig. 1). This suppressive effect was not caused by cytotoxicity of GTE in T cells (Fig. 2).

Previous studies [4, 7] have reported that GTE possessed an anti-cancer activity. GTE showed cytotoxicity in breast cancer cell lines (MDA-MB-231 and MCF-7) and hepatoma cell lines (HepG2 and Hep3B) [4]. The IC_50_ doses of GTE in these susceptible cell lines are 20–30 μg/mL [4]. Our data suggest that PBMCs and Jurkat T cells were resistant to GTE-induced cytotoxicity (Fig. 2).

Ha et al. [12] reported that GTE at a high dose, but not a low dose, inhibited NF-κB activation. In our study, no NF-κB defect in T cell activation was observed with co-treatment at a low concentration of GTE (Fig. 3), although we did find strong inhibition of JNK activity and slight suppression of p38 activity (Fig. 4). LPS treatment induced activation of NF-κB and JNK/p38 [34], it was possible that a low concentration of GTE might also inhibited JNK activity upon LPS treatment in macrophages.

The mechanism by which GTE regulated JNK activity was unclear. One possible mechanism is through the production of reactive oxygen species (ROS) that induce JNK activation to initiate a downstream signal that amplifies ROS production upon stimulation [35]. Previous studies have shown that GTE has an antioxidant activity [4, 5], and the ability of GTE to modulated JNK might be mediated through its activity as a ROS scavenger by blocking the JNK–ROS activation loop.

In adoptive immunotherapy of cancer treatment, AICD of adoptively transferred T cells represents one of the major hurdles to devise an effective immune intervention therapy. Rescuing T cells from AICD might promote anti-tumor immunity [19, 36]. Previous studies have demonstrated that the modulation effect of JNK prevents AICD of adoptively transferred T cells [20, 23]. Our study demonstrated that GTE inhibited JNK activity upon T cell activation and AICD (Figs. 4, 5b). The mechanism of AICD inhibition by GTE might be mediated through modulation of prolonged JNK phosphorylation in activated T cells. In addition to its roles in cell activation and AICD in the immune system, JNK has been shown to be involved in other stress signaling pathways such as those in response to ultraviolet and γ irradiation [37]. Hence, our findings of the effects of GTE on JNK modulation indicate that GTE may have regulatory effects on other cellular responses.

## Conclusion

GTE inhibited T cell activation of Jurkat cells and freshly prepared human PBMCs due to suppression of JNK activity. Furthermore, GTE inhibited AICD by blocking prolonged JNK phosphorylation in activated T cells. Taken together, the anti-inflammatory effects exerted by GTE were mediated via suppression of JNK phosphorylation in T cell activation.

## Additional files

**Additional file 1.** Documentation of permission of research ethic protocol.

**Additional file 2.** Affidavit of approval of Animal Use Protocol.

**Additional file 3.** Minimum standards checklist for confirming the information of methods.

**Additional file 4.** Inhibitory proliferative effect of GTE on PHA-stimulated PBMCs from four individuals. Data are expressed as CPM of 3H-thymidine incorporation and represented as mean ± SD.

## Abbreviations

AICD: activation-induced cell death
CPM: counts per minute
CTLs: cytotoxic T lymphocytes
DAG: diacylglycerol
DMSO: dimethyl sulfoxide
ERK: extracellular signal-regulated kinase
FBS: fetal bovine serum
GT: *Glossogyne tenuifolia*
GTE: *Glossogyne tenuifolia* ethanol extract
HBSS: Hank’s balanced salt solution
HRP: horseradish peroxidase
IκB: nuclear factor of kappa light polypeptide gene enhancer in B-cells inhibitor
IL-2: interleukin-2
IP3: inositol trisphosphate
ITS: internal transcribed spacer
JNK: c-Jun N-terminal kinases
LPS: lipopolysaccharide
MAPKs: mitogen-activated protein kinases
MFI: mean fluorescence intensity
Na_3_VO_4_: sodium orthovanadate
NF-κB: nuclear factor kappa-light-chain-enhancer of activated B cells
PBMCs: peripheral blood mononuclear cells
PBS: phosphate buffered saline
PHA: phytohemagglutinin
PI: propidium iodide
P/I: PMA/ionomycin
PKC: protein kinase C
PMA: 12-*O*-tetradodecanoyl-phorbol-13-acetate
SD: standard deviation
TCR: T cell receptor
