# Supplementary material for: Glossogyne tenuifolia (Hsiang-ju) extract suppresses T cell activation by inhibiting activation of c-Jun N-terminal kinase
Source: Chin Med. 2017 Apr 11;12:9. doi: 10.1186/s13020-017-0130-4 (PMC5387255; doi:10.1186/s13020-017-0130-4)
Supplement: Supplementary file 1 — Additional file 1. Documentation of permission of research ethic protocol. [file 13020_2017_130_MOESM1_ESM.pdf]

國家衛生研究院
醫學研究倫理委員會

Research Ethics Committee
National Health Research Institutes

研究計畫許可書

編號：EC1001101

中華民國 101 年 7 月 4 日
聯絡人：黃俊溢 先生
電話：037-246166 轉 38603

計畫名稱：人類幹細胞影響免疫調節機制之研究
計畫主持人：許素菁助研究員（感染症與疫苗研究所）
本會同意版本內容：
計畫書（版本日期：版本 PB-MDDC-10010-25，日期 100.10.25）
研究計畫申請表（版本日期：版本 1.1，2012/3/28）

計畫執行期間為：2012/6/28~2015/5/31
上述計畫已於 101 年 6 月 28 日經本院醫學研究倫理委員會審查通過。
※ 依照 ICH-GCP 規定，計畫執行每屆滿一年，醫學研究倫理委員會必須重新審查是否繼續進行。本計畫請於 102 年 5 月 28 日前繳交期中報告，以利本會進行審查。若計畫主持人未依本會規定時限內繳交期中報告，本會將不再受理該計畫主持人新計畫之申請。

醫學研究倫理委員會
主任委員

陳立宗

Permission of Research Proposal
National Health Research Institutes

Code: EC1001101

Date: July 4, 2012

Title of the Project: To study the immuno-modulation activity of human stem cells
Principle Investigator: Shu-Ching Hsu, Ph.D.
Contents:
Protocol-- Version and Date: Version PB-MDDC-10010-25, October 25, 2011
Application from-- Version and Date: Version 1.1, March 28, 2012
Approval date: June 28, 2012
Duration of the project: June 28, 2012 to May 31, 2015

※ The constitution and operation of the review board are formulated according to the guidelines of ICH-GCP. The IRB will review your approved project annually. Please send us your Midterm Report before May 28, 2013. If the PI does not send a Midterm Report to the IRB before the requested deadline, the IRB will not process any review application for the PI's new project.

Chairman, Li-Tzong Chen, M.D., Ph.D.
Research Ethics Committee

Li-Tzong Chen
