# Supplementary material for: Glossogyne tenuifolia (Hsiang-ju) extract suppresses T cell activation by inhibiting activation of c-Jun N-terminal kinase
Source: Chin Med. 2017 Apr 11;12:9. doi: 10.1186/s13020-017-0130-4 (PMC5387255; doi:10.1186/s13020-017-0130-4)
Supplement: Supplementary file 2 — Additional file 2. Affidavit of approval of Animal Use Protocol. [file 13020_2017_130_MOESM2_ESM.pdf]

義守大學動物實驗管理小組審查同意書
Affidavit of Approval of Animal Use Protocol
I-Shou University

計畫書編號(IACUC Protocol No):AUP-96-43-007

同意書編號(Approval No):IACUC-ISU-96002

計畫書申請人：方麗雯　　職 稱：助理教授

單 位：醫學營養學系　　飼養及應用地點：實驗動物中心

計 畫 名 稱：香菇有效成分對輔助抗癌及免疫活性調控機制之探討

本「動物實驗計畫書」業經動物實驗管理小組☑實質☐形式審查通過。本計畫預定飼養應用之動物如下:

動物種類	動物數量	飼養及應用期間3
Foxnlnu/Foxnlnu	100 隻	2008 年 8 月 1 日至 2011 年 7 月 31 日
mice/Balb/c	100 隻	2008 年 8 月 1 日至 2011 年 7 月 31 日

The animal use protocol listed below has been reviewed and approved by the Institutional Animal Care and Use Committee (IACUC)

Protocol Title: Study on the mechanisms of *Glossogyne tenuifolia* (L.) Decne on accessory anticancer and immunoregulation

Protocol No: ____________________

Period of Protocol: Valid From: 08/01/2008 To: 07/31/2011

Principle Investigator (PI): Li-Wen Fang

動物實驗管理小組召集人:　　日期: 961224

IACUC Chairman:　　Date:
