# Supplementary material for: Glossogyne tenuifolia (Hsiang-ju) extract suppresses T cell activation by inhibiting activation of c-Jun N-terminal kinase
Source: Chin Med. 2017 Apr 11;12:9. doi: 10.1186/s13020-017-0130-4 (PMC5387255; doi:10.1186/s13020-017-0130-4)

GTE (µg/mL)		0	3.13	6.3	12.5	25	50
Donor 1	Control	944.3 ± 169.7	977 ± 3.5	1046.3 ± 229.1	911 ± 181.7	688 ± 80.6	492.8 ± 91.2
	PHA	45846.3 ± 2899.1	39669 ± 554.4	34967 ± 2350.4	26289.7 ± 907.2	8107.3 ± 359.2	829 ± 529.6
Donor 2	Control	420.3 ± 57.3	388.7 ± 96.9	466 ± 89.1	407.3 ± 89.8	263 ± 113.8	249 ± 120.9
	PHA	56682 ± 200.8	42780 ± 2013.1	37749 ± 6284.8	29339.3 ± 2066.2	9615 ± 376.2	507.3 ± 9.9
Donor 3	Control	451 ± 93.3	415 ± 42.4	445.3 ± 180.3	379.7 ± 66.5	265 ± 101.8	142 ± 38.2
	PHA	111474.3 ± 5941.1	103375 ± 2404.8	91925.7 ± 7929.5	73191 ± 1954.4	32241 ± 2735.8	1244.3 ± 169.7
Donor 4	Control	1874 ± 952.5	1097.7 ± 157.7	1190 ± 246.1	773 ± 147.8	854 ± 101.1	545 ± 155.6
	PHA	68121 ± 299.1	61512.3 ± 1844.8	56309 ± 2728.7	45475.7 ± 1784.0	35311 ± 5321.7	13252 ± 400.2

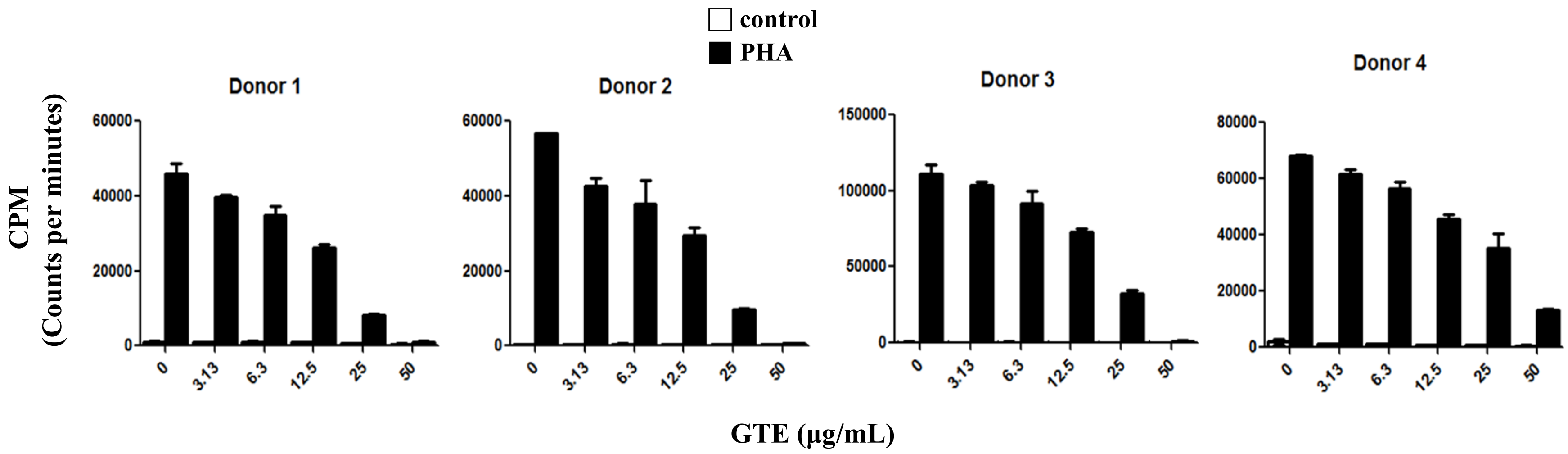

Supplement: Supplementary file 4 — Additional file 4. Inhibitory proliferative effect of GTE on PHA-stimulated PBMCs from four individuals. Data are expressed as CPM of 3H-thymidine incorporation and represented as mean ± SD. [file 13020_2017_130_MOESM4_ESM.pdf]
